# Adaptation and evaluation of the neighborhood environment walkability scale for youth for Chinese children (NEWS-CC)

**DOI:** 10.1186/s12889-021-10530-4

**Published:** 2021-03-11

**Authors:** Gang He, Wendy Huang, Jo Salmon, Stephen H. S. Wong

**Affiliations:** 1 School of Kinesiology and Health, Capital University of Physical Education and Sports, Beijing, China; 2 Department of Sport, Physical Education and Health, Hong Kong Baptist University, Kowloon Tong, Hong Kong, China; 3 Institute for Physical Activity and Nutrition, Deakin University, Melbourne, Australia; 4 Department of Sports Science and Physical Education, The Chinese University of Hong Kong, Shatin, Hong Kong, China

**Keywords:** Physical activity, Built environment, Children, Questionnaire, Validation

## Abstract

**Background:**

The physical activity-environment relationship has been infrequently investigated in Chinese children. Reliable and valid environmental measures specific to the age group and the local context are crucial for better understanding this relationship. The purposes of this study were to adapt the Neighborhood Environment Walkability Scale for youth (NEWS-Y) for Chinese children (termed NEWS-CC), and to examine the reliability and factorial validity of the NEWS-CC.

**Methods:**

The development of the NEWS-CC involved the translation of the NEWS-Y to Chinese and the addition of nine new items capturing Hong Kong specific environmental attributes which were generated in our previous study. A total of 953 Hong Kong children aged 9–14 years volunteered to complete the NEWS-CC twice with 7–14 days apart. Confirmatory factor analysis (CFA) was performed to examine the factorial validity of the NEWS-CC. Test-retest reliability of subscales and individual items in the NEWS-CC was examined by intraclass correlation coefficients (ICCs).

**Results:**

The CFA identified a 7-factor measurement model for the NEWS-CC which fitted the data well, with an additional “pollution” factor not included in the original NEWS-Y. The final NEWS-CC consisted of 67 items in 10 subscales. The test-retest reliability of subscales (range of ICC = 0.47–0.86) and individual items (range of ICC = 0.41–0.79) in the final NEWS-CC was moderate to good.

**Conclusion:**

The results of this study support the psychometric properties of the NEWS-CC. The NEWS-CC can be used to assess physical activity-related neighborhood environment among children in Hong Kong, as well as cities that share similar urban forms with Hong Kong.

**Supplementary Information:**

The online version contains supplementary material available at 10.1186/s12889-021-10530-4.

## Background

The benefits of regular physical activity (PA) [1] and the negative impacts of excessive sedentary behavior (SB) [2] on the health outcomes have been extensively documented for school-aged children. The World Health Organization recommends children to achieve at least 60 min of moderate- to vigorous-intensity PA (MVPA) daily and limit the amount of sedentary time [3]. A meta-analysis with pooled objective data from 20 studies indicated that Chinese children and adolescents spent approximately 41 min/day in MVPA and 530 min/day in SB on average [4]. Based on a recent national survey with a representative sample of Chinese school-aged children, only 13.1% reported to have 60 min of MVPA daily [5]. Longitudinal studies have demonstrated changes of the built environment to be significant determinants of PA behaviors [6]. China has been undergoing rapid urbanization and urban sprawl which may have profound and lasting impacts on residents’ lifestyle, including the PA behaviors of children [7].

Because children have less behavioral autonomy, they are more prone to be affected by the environment of their neighborhood [8]. The impact of the neighborhood built environment on children’s physical activity (PA) has gained research interest in recent years worldwide [9, 10] including China [11]. Reliable and valid environmental measures are crucial for better understanding the relationship between the built environment and PA in children. The built environment has been measured mainly using three methods; perceived measures, direct observation (audits), and Geographic Information Systems (GIS) [12]. Audits and GIS are objective measures and are recommended by researchers for accurately assessing the built environment [13]. However, compared to objective measures, perceived measures are valuable in capturing different attributes of neighborhood environment that are predictive of children’s PA [8]. In addition, perceptions about the environment are more amenable to change than the actual environment [14]. Moreover, the relationship between physical activity and the built environment can vary depending on whether the environmental measure is objective or subjective [15, 16]. Therefore, having an understanding of the target population’s perceptions of their neighborhood built environment could better inform the development of specific interventions targeting corresponding environmental correlates.

Several instruments assessing children’s perceptions of their neighborhood environment have been developed in recent years, and have shown acceptable reliability [17, 18]. However, these instruments provide less comprehensive measures of the neighborhood built environment in contrast to the framework developed by Pikora and colleagues [19]. This framework provides a comprehensive system of guidance for understanding a PA-related environment which identified four main environmental domains (functional, safety, aesthetic, and destination) comprising nine environmental elements (walking surface, streets, traffic, permeability, personal safety, traffic safety, streetscape, views, and facilities) [19].

Among existing questionnaires assessing the perceived neighborhood environment, the Neighborhood Environment Walkability Scale (NEWS) and its abbreviated version (NEWS-A) provide by far the most comprehensive measures of the PA-related neighborhood environment and are the most frequently used internationally [20, 21]. The NEWS and the NEWS-A consist of 68 and 54 items respectively measuring eight environmental elements in the neighborhood, i.e., residential density, land use mix-diversity, aesthetics, land use mix-access, street connectivity, walking facilities, crime safety, and traffic safety [22, 23]. A version of the NEWS adapted to youth (NEWS-Y) was developed from the NEWS-A by adding items relevant to youth and an additional checklist of 14 recreational facilities related to PA of youth [24]. The NEWS-Y showed a good test-retest reliability in a sample of adolescents in the USA [24]. A recent study further adapted NEWS-Y to accommodate its application among 15 countries and regions including Hong Kong within the International Physical Activity and Environment Network adolescent project (NEWS-Y-IPEN), and the NEWS-Y-IPEN demonstrated good factorial and construct validity [25].

Perception of environments depends on past experience, aspiration levels, adaptation processes and individual personality [12], thus measures of perceived environments should be specific to age groups, culture, and local environmental attributes. Children have unique understanding of their experiences, and children at 8–11 years can provide reliable reports on questionnaires developed especially for this age group [26].

Huang and colleagues have modified the NEWS to assess perceptions of the neighborhood environment in children, but this instrument only adopted 11 selective items on aesthetics, convenience, safety, and 22 items on accessibility from the NEWS and provided limited measures of the perceived environment [27]. To the best of our knowledge, NEWS-Y has not been adapted and evaluated specifically for children. Therefore, the purpose of this study was to adapt the NEWS-Y for Chinese children (termed NEWS-CC) and test its reliability and factorial validity in a sample of Hong Kong children.

## Methods

This study was conducted in three steps: (1) instrument development; (2) pilot testing; (3) reliability and factorial validity testing.

### Participants and procedures

#### Instrument development

The source instrument used to develop the NEWS-CC was the original English version of the NEWS-Y [24]. The NEWS-Y consists of 67 items grouped into subscales measuring nine aspects of the neighborhood environment including land use mix-diversity, traffic safety, crime safety, aesthetics, walking/cycling facilities, street connectivity, land use mix-access, residential density, and recreational facilities. All subscales use a 4-point Likert scale, except for land use mix-diversity, recreational facilities, and residential density. Land use mix-diversity and recreational facilities are checklists of proximity of diverse destinations and facilities in the neighborhood. Residential density was assessed by four items asking about residence types in the neighborhood using a 5-point scale. The development of the NEWS-CC involved translation and adaptation of items to capture Hong Kong-specific environmental attributes, such as covered sidewalks, bridge or tunnel help to cross the street, and garden on the roof or platform of buildings.

The translation of the NEWS-Y from English to Chinese (Cantonese) followed the process recommended by the World Health Organization [28]: First, the original English NEWS-Y was independently translated into Chinese (forward translation) by two bilingual Chinese native speakers. The academic backgrounds of the two forward translators are exercise science and geography, respectively. Second, these two independent translations were synthesized by the forward translators with comparison to the original English questionnaire. Inconsistent translations were discussed and modified one by one and a synthesized Chinese translation was formed as a result. Third, the synthesized Chinese translation was translated into English (back translation) by another translator (back translator) who is a bilingual Chinese native speaker with the background of exercise science. Fourth, one of the forward translators, the back translator and another English native speaker with the background of exercise science evaluated the semantic equivalence (meaning of each item remains the same after translation into a different language) between the English back translation and the original English questionnaire using the Flaherty’s 3-Point Scale [29]. Each evaluation panel member independently gave a score of 1–3 for each item in the back translation compared with the original version, with score 3 indicating exactly the same meaning in both versions, score 2 indicating almost the same meaning in both versions, and score 1 indicating a different meaning in each version. No translated items received a uniform score of “1”. Items receiving mixed scores of “1” to “3” were re-worked until equivalence was achieved.

The adaptation of the NEWS-Y (thereafter, the NEWS-CC) included the modification of the residential density subscale and the addition of nine new items. The 4-item residential density subscale in the NEWS-Y was substituted by a 6-item subscale developed by Cerin and colleagues which has been used to measure perceived residential density of Hong Kong [20]. In order to identify items specific to children and the Hong Kong context, 34 children aged 10–11 years from four types of neighborhoods varying in socio-economic status (SES) and walkability in Hong Kong were interviewed using Nominal Group Technique (detailed procedures are reported elsewhere [30]. A total of nine novel items deemed to be highly relevant to children’s PA were integrated into corresponding subscales of the NEWS-Y: six items to the aesthetics subscale (i.e. *dogs/animals fouling*, *garden on the roof or platform of buildings*, *air is fresh in my neighborhood*, *smokers in the streets*, *fumes from restaurants*, and *noise from worksite*); one item to the walking facility subscale (i.e. *sidewalk with covers*); one item to the crime safety subscale (i.e. *people make me feel unsafe*); and one item to the traffic safety subscale (i.e. *bridge or tunnel help to cross the street*). Therefore, the initial NEWS-CC consisted of 78 items in nine subscales.

#### Pilot testing

The newly developed NEWS-CC was pilot-tested among a convenience sample of 66 children in grade 5 or grade 6 from two primary schools. The children were required to mark the items that they could not understand or felt unacceptable while they were completing the questionnaire. The researcher afterwards discussed with the children who made marks on their questionnaire to obtain detailed opinions of the children regarding those items, and the children were also encouraged to suggest alternative wording. All children completed the NEWS-CC and socio-demographic questions within 25 min, which revealed the feasibility of administering the questionnaire within one primary school lesson (normally 35 min in Hong Kong). A total of 17 unclear items were mentioned by the children. Rewordings of the unclear items were subsequently made based on the results of the pilot testing.

#### Participants

A total of 1067 children in grades 4 to 6 were recruited from seven primary schools located in different districts in Hong Kong of varying SES. The children completed the NEWS-CC twice, 7–14 days apart. Children were also required to report their home address to identify the Tertiary Planning Unit (TPU) in which their home was located. The TPU system was developed by Hong Kong Planning Department for the purpose of town planning. A TPU is the smallest census-based geographic unit used in Hong Kong. Hong Kong is divided into 289 TPUs. Considering data precision, a TPU with less than 1000 persons is merged with adjacent TPU(s). A TPU has an average area of 5.34 km^2^ and the average number of households and residents per TPU are 11,441 and 34,151, respectively [31].

This study was approved by the Survey and Behavioral Research Ethics Committee of The Chinese University of Hong Kong. The study protocol was in accordance with the Declaration of Helsinki. Informed consent was obtained from all participants.

## Data analysis

To examine the factorial validity of the initial NEWS-CC, a confirmatory factor analysis (CFA) was conducted on the 38 items from the six subscales which use the same 4-point Likert scale: aesthetics, land use mix-access, street connectivity, walking facilities, crime safety, and traffic safety. The other subscales (and 40 items), i.e. land use diversity, recreational facilities, and residential density, were not appropriate for CFA because (1) they are either a check list of diverse destinations and facilities (subscales of land use diversity and recreational facilities), or are used for a formula to calculate residential density (the subscale of residential density), so they do not load on a latent factor; (2) the response formats of items in these subscales are different from those in other subscales. Due to the hierarchical nature of the data (individuals nested in TPUs), intraclass correlation coefficients (ICCs) were calculated for each NEWS-CC item to determine whether a multilevel CFA or a single-level CFA should be conducted. An ICC value larger than 0.10 indicates the need to do the multilevel CFA [23].

The CFA was conducted in two steps: first, the fit of the a priori model for the NEWS-CC was examined. The a priori model of the NEWS-CC adopted the six-factor structure of the original NEWS-Y [24]; second, the a priori model of the NEWS-CC was respecified if it could not meet the criteria for an acceptable model fit, i.e. no less than 0.90 for the comparative fit index (CFI), the nonnormed fit index (NNFI), and the goodness-of-fit index (GFI); no larger than 0.10 for the standardized root mean squared residual (SRMR); and no larger than 0.06 for the root mean square error of approximation (RMSEA) [32]. The 90% confidence intervals (90% CIs) of the RMSEA were also reported [33]. Respecifications of the models were guided by the LMTEST and Wald Test at the item level with consideration of the theoretically meaningful interpretation of the respecification. The CFA was conducted using EQS. 6.1 (Multivariate Software Inc., 2004).

One-way model single-measure intraclass correlation coefficients (ICCs) were calculated to examine the test-retest reliability of subscales and those individual items comprising the subscale. The interpretation of ICC values for reliability was: 0.00–0.20 as slight, 0.21–0.40 as fair, 0.41–0.60 as moderate, 0.61–0.80 as substantial, and 0.81–1.00 as almost perfect reliability [34]. Items with a test-retest ICC value of 0.40 or less were excluded from the respecified NEWS-CC, resulting in the final NEWS-CC. The CFA was performed on the final NEWS-CC to re-examine its factorial validity.

## Results

The final analytic sample comprised 953 children who provided complete questionnaire data twice, among which 426 were boys (mean age = 11.2 years, SD = 1.0) and 527 were girls (mean age = 10.8 years, SD = 1.0).

### Confirmatory factor analysis of the original NEWS-CC

The mean ICC value of the NEWS-CC items was 0.06 (SD = 0.07), and only three of 38 factor-analyzable items had an ICC value larger than 0.10. This indicated that a single-level rather than a multilevel CFA should be conducted. Meanwhile, the CFA was performed using the within-TPU covariance matrix to control possible between-TPU effects. A maximum likelihood estimation was used in the CFA as the univariate skewness (< 2.0) and univariate kurtosis values (< 7.0) of items were all within an acceptable level [35].

The results of the CFA are shown in Table 1. The a priori model of the original NEWS-CC consisted of 38 items loading on six latent factors (i.e. aesthetics, land use mix-access, street connectivity, walking facilities, crime safety, and traffic safety). The fit of the a priori model of the NEWS-CC was poor with no model fit index being met. The respecified model indicated a seven-factor structure with an additional “pollution” factor being added and a within-factor error covariance modeled. Nine items were excluded from the a priori model, i.e. *dogs/animals fouling*, *difficult to park in shopping malls*, *hilly streets*, *walking barriers*, *high crime rate*, *too many vehicles*, *low traffic speed*, *speeding drivers*, and *fresh air*. The model fit of the respecified NEWS-CC was acceptable in terms of the model fit indices of GFI, CFI, SRMR, and RMSEA being met and the NNFI achieving a marginal level (Table 1). For both models examined, no cross-loading of items on factors was modeled.

**Table 1:** Results of CFA of the NEWS-CC

Model	χ^**2**^	df	GFI	CFI	NNFI	SRMR	RMSEA (90%CI)
A priori NEWS-CC	3039.2	687	0.78	0.66	0.63	0.087	0.064 (0.062–0.067)
Respecified NEWS-CC	872.0	355	0.93	0.91	0.89	0.053	0.042 (0.038–0.045)

### Test-retest reliability of the respecified NEWS-CC

The respecified NEWS-CC consisted of 10 subscales and 69 items. The results of the test-retest reliability of the respecified NEWS-CC are shown in Table 2. At the item level, most items showed moderate to substantial levels of reliability (range of ICC = 0.41–0.79). Two items with poor reliability (*E3 easy to walk to public transport*, ICC = 0.33 and *F2 short distance between street crossings*, 0.18) were excluded in the final NEWS-CC. All subscales in the final NEWS-CC indicated moderate to almost perfect test-retest reliability with the ICC ranging from 0.47 to 0.86.

**Table 2:** Test-retest ICCs^a^ of subscales and individual items of the respecified NEWS-CC

Subscale / Item	ICC (95%CI)
**A. Land use mix-diversity**	**0.86 (0.84–0.88)**^**b**^
1. convenience store	0.61 (0.56–0.66)
2. super market	0.68 (0.64–0.72)
3. hardware store	0.76 (0.73–0.79)
4. public market	0.74 (0.71–0.77)
5. laundry	0.70 (0.66–0.73)
6. clothing store	0.71 (0.67–0.74)
7. post office	0.77 (0.74–0.80)
8. library	0.79 (0.76–0.82)
9. primary school	0.74 (0.71–0.77)
10. secondary school	0.71 (0.68–0.75)
11. bookstore	0.67 (0.63–0.70)
12. fast food restaurant	0.68 (0.64–0.72)
13. coffee shop	0.67 (0.63–0.71)
14. bank/finance center	0.62 (0.58–0.66)
15. non-fast food restaurant	0.61 (0.57–0.65)
16. video store	0.67 (0.63–0.71)
17. pharmacy	0.72 (0.69–0.75)
18. hair salon	0.72 (0.68–0.75)
19. any office/construction site	0.59 (0.54–0.63)
20. bus stop/MTR	0.55 (0.50–0.60)
**B. Recreational facilities**	**0.80 (0.77–0.83)**^**b**^
1. Indoor leisure or sport facilities	0.50 (0.45–0.55)
2. Beach, lake, river or creek	0.62 (0.58–0.66)
3. Cycling/ hiking/ walking trail	0.57 (0.52–0.62)
4. Basketball court	0.71 (0.67–0.74)
5. Other playground/ sports field	0.60 (0.55–0.64)
6. YMCA	0.63 (0.59–0.67)
7. Youth Societies	0.62 (0.58–0.66)
8. Swimming pool	0.76 (0.73–0.79)
9. Jogging trail	0.65 (0.60–0.69)
10. Schools with sports fields open to public	0.48 (0.43–0.53)
11. Sitting-out area	0.52 (0.47–0.57)
12. Parks	0.64 (0.60–0.68)
13. Children’s playground	0.66 (0.62–0.70)
14. Open places (grass, sands, clay)	0.60 (0.55–0.64)
**C. Residential density**	**0.66 (0.62–0.70)**^**c**^
1. detached single-family houses	0.59 (0.54–0.63)
2. multi-family houses (1–3 stories)	0.64 (0.60–0.68)
3. apartments with 4–6 stories	0.62 (0.58–0.66)
4. apartments with 7–12 stories	0.58 (0.54–0.63)
5. apartments with 13–20 stories	0.67 (0.63–0.70)
6. apartments with > 20 stories	0.72 (0.69–0.75)
**D. Aesthetics**	**0.62 (0.57–0.66)**^**b**^
1. trees	0.56 (0.52–0.61)
2. interesting things	0.53 (0.48–0.58)
3. beautiful natural sceneries	0.60 (0.55–0.64)
4. beautiful buildings	0.54 (0.49–0.59)
5. gardens on roof of buildings	0.52 (0.47–0.57)
**E. Land use mix-access**	**0.56 (0.50–0.60)**^**b,d**^
1. easy to walk to shops	0.51 (0.46–0.56)
2. easy to go to various places	0.41 (0.18–0.59)
*3. easy to walk to public transport*	*0.33 (0.09–0.53)*
**F. Street connectivity**	**0.47 (0.25–0.64)**^**b,d**^
1. not many cul-de-sac	0.41 (0.19–0.59)
*2. short distance between street crossings*	*0.18 (−0.07–0.41)*
3. many alternative routes	0.54 (0.34–0.69)
**G. Walking facility**	**0.54 (0.34–0.70)**^**b**^
1. sidewalks on most streets	0.43 (0.21–0.61)
2. sidewalks separated by parked cars	0.54 (0.34–0.69)
3. sidewalks separated by barriers or grass	0.69 (0.53–0.80)
4. covered sidewalks	0.64 (0.47–0.77)
**H. Crime safety**	**0.68 (0.63–0.71)**^**b**^
1. unsafe to be outdoors in evenings	0.41 (0.35–0.47)
2. afraid of being outside alone near home	0.59 (0.54–0.63)
3. afraid of being outside with friends near home	0.56 (0.51–0.61)
4. afraid of being outside on nearby streets	0.57 (0.52–0.61)
5. afraid of being outside in nearby parks	0.60 (0.55–0.64)
6. people make me feel unsafe	0.47 (0.42–0.52)
**I. Traffic safety**	**0.52 (0.47–0.57)**^**b**^
1. good lighting in evenings	0.42 (0.36–0.48)
2. easy to see pedestrians or cyclists	0.60 (0.41–0.73)
3. crosswalks or signals	0.50 (0.45–0.55)
4. bridges or tunnels	0.54 (0.49–0.59)
**J. Pollution**	**0.69 (0.65–0.73)**^**b**^
1. much exhaust gas	0.54 (0.49–0.59)
2. smokers in streets	0.54 (0.49–0.59)
3. fume from restaurants	0.50 (0.44–0.55)
4. noise from worksite	0.58 (0.54–0.63)

^a^: test-retest ICCs were calculated by one-way model single-measure intraclass correlation coefficients;

^b^: computing based on average score of items in the subscale

^c^: computing based on the formula of (C1 + C2*12 + C3*25 + C4*50 + C5*75 + C6*100) [20]

^d^: the ICC value of subscale E (Land use mix-access) was calculated based on items E1 and E2; the ICC value of subscale F (Street connectivity) was calculated based on items F1 and F3

### Confirmatory factor analysis of the final NEWS-CC

The full version of the final NEWS-CC therefore contained 67 items in 10 subscales including land use mix-diversity (20 items), recreational facilities (14 items), residential density (6 items), aesthetics (5 items), land use mix-access (2 items), street connectivity (2 items), walking facilities (4 items), crime safety (6 items), traffic safety (4 items), and pollution (4 items). The CFA was conducted on the 27 items in the subscales of aesthetics, land use mix-access, street connectivity, walking facilities, crime safety, traffic safety, and pollution to re-examine the factorial validity of the final NEWS-CC.

The model fit of the final NEWS-CC was better than the respecified NEWS-CC in that all of the five model fit indices were met (GFI = 0.94; CFI = 0.92; NNFI = 0.91; SRMR = 0.053; RMSEA = 0.042, 90%CI: 0.038–0.045; χ^2^(302) = 735.2). In the model of the final NEWS-CC, one within-factor error covariance was included, whereas no cross-loading of items on factors was modeled. As each latent factor measured a certain aspect of the neighborhood environment believed to impact PA, these factors were modeled as inter-correlated. As shown in Table 3, moderate correlations were observed between factors of aesthetics and street connectivity, land use mix-access and street connectivity, street connectivity and walking facilities, land use mix-access and traffic safety, street connectivity and traffic safety, and walking facilities and traffic safety.

**Table 3:** Correlations between latent factors of the final NEWS-CC

Factors	Factor 1 (aesthetics)	Factor 2 (land use mix-access)	Factor 3 (street connectivity)	Factor 4 (walking facilities)	Factor 5 (crime safety)	Factor 6 (traffic safety)	Factor 7 (pollution)
**Factor 1**	1						
**Factor 2**	0.32	1					
**Factor 3**	***0.43***	***0.77***	1				
**Factor 4**	0.37	0.24	***0.54***	1			
**Factor 5**	−0.11	0.10	0.01	−0.07	1		
**Factor 6**	0.38	***0.45***	***0.56***	***0.74***	−0.14	1	
**Factor 7**	0.26	−0.08	−0.08	− 0.03	0.19	− 0.02	1

## Discussion

This study adapted the NEWS-Y for Chinese children (termed NEWS-CC), and tested the reliability and factorial validity of the NEWS-CC in a sample of Hong Kong children. A 7-factor measurement model for the NEWS-CC was identified. The test-retest reliability of subscales and individual items in the final NEWS-CC was moderate to good.

### Development of the NEWS-CC

Previous studies in Hong Kong have also identified similar environmental attributes that potentially affect PA in adults and seniors, such as aesthetics-related items *dogs/animals fouling*, *air pollution*, and *noise pollution*, a walking facility-related item *covered sidewalks*, crime safety-related items *homeless people* and *drug addicts and/or prostitutes in neighborhood*, and a traffic safety-related item *bridge or tunnel help to cross the street* [20, 36]. *Garden on the roof or platform of buildings*, a common Hong Kong attribute due to the high density of buildings in Hong Kong, was an attribute that was not addressed in previous studies.

### Factorial validity of the NEWS-CC

The a priori model of the NEWS-CC hypothesized a six-factor structure defined by the NEWS-Y and the fit of the a priori model was poor. The final model of the NEWS-CC identified a seven-factor structure with the addition of a “pollution” factor and the exclusion of 11 items compared to the a priori model.

Several studies have investigated the factorial structure of different versions of the NEWS in several countries/regions [20, 23, 32, 37, 38]. Multilevel CFA has established a six-factor structure at the individual level and a five-factor structure at the census block level of the NEWS and the NEWS-A in American adults [23, 37]. A similar six-factor structure was confirmed among female seniors in America [38, 39]. A slightly different measurement model was identified for the Australian version of the NEWS (7 factors at the individual level and 5 factors at the census block level) [32]. In contrast to the factorial structure of the NEWS established in the Western countries, a 12-factor structure was identified for the adapted NEWS in Hong Kong seniors [20]. The inconsistency of measurement models of different versions of the NEWS indicates the necessity of establishing the factorial validity of the NEWS in different locations and age groups. To facilitate between-country comparisons, some studies have attempted to establish country-specific measurement models of NEWS among adults [40] and adolescents [25]. Yet in children, no study has examined the factorial structure of NEWS specifically adapted for this age group.

The final measurement model of the NEWS-CC encompassed seven latent factors including six factors that overlapped with those in the NEWS-Y [24] and one unique pollution factor. Construction of the pollution factor was due to the weak association of four items (i.e. *smokers in the streets*, *fume from restaurants*, *noise from worksite* and *much exhaust gas*) with hypothesized aesthetics or traffic safety factors, and the relevance of the meaning of these four items to air or noise pollution. Air and noise pollution has also been previously indicated to be a distinct environmental attribute shared by East Asian ultra-dense cities such as Hong Kong [36].

A total of 11 items were excluded from the a priori model of the NEWS-CC. For the three items that originally loaded on the land use mix-access factor (i.e. *difficult to park in shopping malls*, *hilly streets*, and *walking barriers*), a previous validation study of the NEWS in adults similarly demonstrated their low factor loadings on the land use mix-access factor at the individual level [23]. Subsequent validation studies of the NEWS in adults in the USA and in Australia also treated these items as single items at the individual level and indicated their independence from the land use mix-access factor at the census block level [32, 37]. As children do not drive cars and access to shops is easier for Hong Kong children via public transport, it is easy to understand that parking difficulties won’t influence their perceptions of accessibility in this study. The terrain of Hong Kong is extremely hilly with its elevation ranging from 0 m to 957 m (Hong Kong Geographic Data. Lands Department of Hong Kong), so the presence of hilly streets and of stairs is very common in Hong Kong [20, 36]. However, due to the well-developed pedestrian infrastructures that helps children overcome walking difficulties [41], hilly streets as well as other potential walking barriers (e.g., worksites, railway lines, and rivers) do not necessarily impact children’s access to services.

As to items hypothesized to load on the traffic safety factor (i.e. *too many vehicles*, *low traffic speed*, and *speeding drivers*), the independence of items measuring traffic speed (i.e. *low traffic speed* and *speeding drivers*) from other items in the traffic safety factor has been previously demonstrated in Hong Kong [20], and independence of traffic load from the traffic safety-related items has been documented in Australia [32]. Hong Kong has a very heavy traffic load compared to many other territories. Therefore, infrastructures such as pedestrian crossings, traffic signals, and bridges or tunnels, which effectively help pedestrians to be free from traffic hazards, are very popular in Hong Kong. Thus, although traffic load and traffic speed are concerns for children and parents, given the highly developed pedestrian infrastructures, traffic load and speed features might not impact children’s perceived traffic safety as much as may otherwise be expected. Another reason might be due to children not being able to provide accurate estimations of the “load” or “speed” concepts.

In contrast to existing studies that have established factor structure of the NEWS in adults [20, 23, 37], *high crime rate* was found to be independent from crime-related items in this study. This item, contrary to other crime-related items in the NEWS-CC, was asking about a general “impression” of the crime nearby. Children have more limited access to the media that may report information of crime (e.g., TV, internet, newspaper) than adults. Therefore, children may not have a clear perception of crimes that may have happened nearby. The crime rate, although still being a concern, was not perceived by children as a safety issue that impacted their PA behaviors.

Being a newly added item, *dogs/animals fouling* has been previously identified as a unique environmental attribute of Hong Kong [20, 36] and was hypothesized to load on the aesthetics factor. However, this item was shown to be less relevant to aesthetics in this study. The independence of animal fouling from other natural or built aesthetics was supported by a study among Hong Kong seniors [20]. As a matter of fact, dogs/animals fouling does not necessarily co-occur with natural or built aesthetics. *Fresh air* is another newly added item that was found not to load on the hypothesized aesthetics factor or the newly identified pollution factor. This item was asking about children’s general perception of air quality and was not as visual and vivid as other items measuring aesthetics or pollution in the NEWS-CC. In addition, due to the limited residential area of Hong Kong, the variability of responses to the general impression of the air quality was low compared to other aesthetics- or pollution-related items in the NEWS-CC.

### Test-retest reliability of the NEWS-CC

The reliability test revealed poor reliability of two items (i.e., *easy to walk to public transport* and *short distance between street crossings*). The low reliability of these two items might be attributed to their low response variability (SD = 0.63 and 0.65 respectively), as it was indicated that restricted variability may lead to very low ICC values [20]. Due to the compact streets and developed public transport system of Hong Kong [41], it is not surprising that the response variabilities to these two items were low.

Six items in subscales E to I showed relatively low ICC values (i.e., between 0.4 and 0.5). Three items (i.e., *not many cul-de-sac, sidewalks on most streets,* and *good lighting in evenings*) are pertaining to children’s impression of objective surrounding environment. Owing to the well-built street network and infrastructure in general, children had homogenous high response to these three items (mean scores ranging from 3.14 to 3.23) thus possibly reducing the ICC values. The other three items (i.e., *easy to go to various places, unsafe to be outdoors in evenings,* and *people make me feel unsafe*) are pertaining to children’s subjective feelings. Meaning of “various places” and feeling of “unsafe” may not be clear enough to children. Even though children can understand the question itself, they may not be able to give stable responses to these items. These items could be re-worked to improve response reliability and further examined in more diverse urban environments in future research.

At the subscale level, the overall reliability of the final NEWS-CC in the examined sample was moderate to good (ICC of subscales ranging from 0.47 to 0.86). These results are similar to those reported for the NEWS-Y in American adolescents (ICC of subscales ranging from 0.56 to 0.87) [24]. This supported the psychometric appropriateness of using this newly developed instrument to measure the PA-related neighborhood environment for Hong Kong children. Relative to other versions of the NEWS examined in Hong Kong adults and seniors, the reliability of the NEWS-CC was slightly higher than the NEWS-CS in Hong Kong seniors (ICC of subscales ranging from 0.37 to 0.77) [20], whereas the reliability of both the NEWS-CC and the NEWS-CS was lower than that reported for the Chinese NEWS-A in Hong Kong adults (ICC of subscales ranging from 0.57 to 0.99) [42], indicating that age-related cognitive abilities may contribute to the observed decline of response reliability [20]. Compared to the NEWS-CC, a higher level of reliability (ICC of subscales ranging from 0.84 to 0.89) was observed in a similar measurement of the neighborhood environment in Hong Kong children (aged 9–14 years) using a similar between-administration interval (10 days) and the same self-administered method [18]. However, this instrument used a shorter measure of the neighborhood environment consisting of only ten items in two subscales, which suggested that the length of the questionnaire may potentially impact the response reliability [43].

### Limitations

The present study has some limitations. First, urban forms vary across China cities in spacial scale, population, economics, public services, infrastructure, and etc. [44]. The results of this study may not be generalized to other places that have disparate urban forms. Second, concurrent validity of the NEWS-CC was not examined in this study. Whether perceived environment assessed by NEWS-CC reflects the environment correlated with children’s PA behavior need to be further investigated by future research. Third, although the NEWS-CC was adapted specifically for children, relatively low response reliabilities were observed for some NEWS-CC items. This might be attributed to children’s limit cognitive ability in fully understanding this lengthy questionnaire. Future research may consider to develop an abbreviated version of the NEWS-CC which could be more reader-friendly to children and has the potential to improve the response reliability.

## Conclusions

The newly developed NEWS-CC possesses adequate factorial validity and test-retest reliability in a sample of Chinese children. The results of this study provide evidence of psychometric properties that support the NEWS-CC to assess PA-related neighborhood environment among children in China cities that share similar urban forms with Hong Kong. Concurrent validity of the NEWS-CC needs to be further examined in future studies.

## Supplementary Information

**Additional file 1.**

**Additional file 2.**

## Data Availability

The datasets used and/or analyzed during the current study are available from the corresponding author on reasonable request.
