# Supplementary material for: Adaptation and evaluation of the neighborhood environment walkability scale for youth for Chinese children (NEWS-CC)
Source: BMC Public Health. 2021 Mar 11;21:480. doi: 10.1186/s12889-021-10530-4 (PMC7949240; doi:10.1186/s12889-021-10530-4)
Supplement: Supplementary file 1 — Additional file 1. [file 12889_2021_10530_MOESM1_ESM.docx]

**兒童身體活動相關社區環境調查問卷**

**A．你屋企附近商店、食肆及其他公共場所**

你從屋企到最近的商店或下列地方需要步行多久？請圈出你步行到每個地方的時間，即使你不常去。

| 1 | 便利店/士多（如7-11） | 1-5分鐘 | 6–10分鐘 | 11-20分鐘 | 21-30分鐘 | 31+分鐘 | 不知道 |
| --- | --- | --- | --- | --- | --- | --- | --- |
| 2 | 超級市場 | 1-5分鐘 | 6–10分鐘 | 11-20分鐘 | 21-30分鐘 | 31+分鐘 | 不知道 |
| 3 | 五金鋪 | 1-5分鐘 | 6–10分鐘 | 11-20分鐘 | 21-30分鐘 | 31+分鐘 | 不知道 |
| 4 | 街市 | 1-5分鐘 | 6–10分鐘 | 11-20分鐘 | 21-30分鐘 | 31+分鐘 | 不知道 |
| 5 | 洗衣店或乾洗店 | 1-5分鐘 | 6–10分鐘 | 11-20分鐘 | 21-30分鐘 | 31+分鐘 | 不知道 |
| 6 | 服裝店 | 1-5分鐘 | 6–10分鐘 | 11-20分鐘 | 21-30分鐘 | 31+分鐘 | 不知道 |
| 7 | 郵局 | 1-5分鐘 | 6–10分鐘 | 11-20分鐘 | 21-30分鐘 | 31+分鐘 | 不知道 |
| 8 | 圖書館 | 1-5分鐘 | 6–10分鐘 | 11-20分鐘 | 21-30分鐘 | 31+分鐘 | 不知道 |
| 9 | 小學 | 1-5分鐘 | 6–10分鐘 | 11-20分鐘 | 21-30分鐘 | 31+分鐘 | 不知道 |
| 10 | 中學 | 1-5分鐘 | 6–10分鐘 | 11-20分鐘 | 21-30分鐘 | 31+分鐘 | 不知道 |
| 11 | 書店 | 1-5分鐘 | 6–10分鐘 | 11-20分鐘 | 21-30分鐘 | 31+分鐘 | 不知道 |
| 12 | 快餐店（如麥當勞、大家樂） | 1-5分鐘 | 6–10分鐘 | 11-20分鐘 | 21-30分鐘 | 31+分鐘 | 不知道 |
| 13 | 咖啡店（如星巴克） | 1-5分鐘 | 6–10分鐘 | 11-20分鐘 | 21-30分鐘 | 31+分鐘 | 不知道 |
| 14 | 銀行/財務中心 | 1-5分鐘 | 6–10分鐘 | 11-20分鐘 | 21-30分鐘 | 31+分鐘 | 不知道 |
| 15 | 非快餐店（餐廳/酒樓/茶餐廳） | 1-5分鐘 | 6–10分鐘 | 11-20分鐘 | 21-30分鐘 | 31+分鐘 | 不知道 |
| 16 | 影音光碟店 | 1-5分鐘 | 6–10分鐘 | 11-20分鐘 | 21-30分鐘 | 31+分鐘 | 不知道 |
| 17 | 藥房 | 1-5分鐘 | 6–10分鐘 | 11-20分鐘 | 21-30分鐘 | 31+分鐘 | 不知道 |
| 18 | 髮廊/理髮店 | 1-5分鐘 | 6–10分鐘 | 11-20分鐘 | 21-30分鐘 | 31+分鐘 | 不知道 |
| 19 | 寫字樓/地盤 | 1-5分鐘 | 6–10分鐘 | 11-20分鐘 | 21-30分鐘 | 31+分鐘 | 不知道 |
| 20 | 小巴/巴士/港鐵站 | 1-5分鐘 | 6–10分鐘 | 11-20分鐘 | 21-30分鐘 | 31+分鐘 | 不知道 |

**B．你屋企附近的休閒場所**

你從屋企到最近的下列休閒場所需要步行多久？請圈出你步行到每個地方的時間，即使你不常去。

| 1 | 室內休閒或運動設施（公眾或私人） | 1-5分鐘 | 6–10分鐘 | 11-20分鐘 | 21-30分鐘 | 31+分鐘 | 不知道 |
| --- | --- | --- | --- | --- | --- | --- | --- |
| 2 | 海灘、湖、河或小溪 | 1-5分鐘 | 6–10分鐘 | 11-20分鐘 | 21-30分鐘 | 31+分鐘 | 不知道 |
| 3 | 單車/行山/步行徑 | 1-5分鐘 | 6–10分鐘 | 11-20分鐘 | 21-30分鐘 | 31+分鐘 | 不知道 |
| 4 | 籃球場 | 1-5分鐘 | 6–10分鐘 | 11-20分鐘 | 21-30分鐘 | 31+分鐘 | 不知道 |
| 5 | 其他運動場地/球場（如足球、橄欖球、壘球、網球、滑板/溜冰） | 1-5分鐘 | 6–10分鐘 | 11-20分鐘 | 21-30分鐘 | 31+分鐘 | 不知道 |
| 6 | YMCA（基督教青年會） | 1-5分鐘 | 6–10分鐘 | 11-20分鐘 | 21-30分鐘 | 31+分鐘 | 不知道 |
| 7 | 青少年協會 (如小童群益會) | 1-5分鐘 | 6–10分鐘 | 11-20分鐘 | 21-30分鐘 | 31+分鐘 | 不知道 |
| 8 | 游泳池 | 1-5分鐘 | 6–10分鐘 | 11-20分鐘 | 21-30分鐘 | 31+分鐘 | 不知道 |
| 9 | 緩跑徑 | 1-5分鐘 | 6–10分鐘 | 11-20分鐘 | 21-30分鐘 | 31+分鐘 | 不知道 |
| 10 | 有運動場所開放給公眾的學校 | 1-5分鐘 | 6–10分鐘 | 11-20分鐘 | 21-30分鐘 | 31+分鐘 | 不知道 |
| 11 | 休憩公園 | 1-5分鐘 | 6–10分鐘 | 11-20分鐘 | 21-30分鐘 | 31+分鐘 | 不知道 |
| 12 | 大公園 | 1-5分鐘 | 6–10分鐘 | 11-20分鐘 | 21-30分鐘 | 31+分鐘 | 不知道 |
| 13 | 遊樂場 | 1-5分鐘 | 6–10分鐘 | 11-20分鐘 | 21-30分鐘 | 31+分鐘 | 不知道 |
| 14 | 空地（草地、沙/土地） | 1-5分鐘 | 6–10分鐘 | 11-20分鐘 | 21-30分鐘 | 31+分鐘 | 不知道 |

**C．你屋企附近的住宅類型**

「屋企附近」指的是從你屋企出發步行10-15分鐘可以到達的範圍。

| 1. | 你屋企附近有單一家庭的獨立住宅嗎（如別墅、村屋）？ | | | | |
| --- | --- | --- | --- | --- | --- |
|  | 1 沒有 | 2 很少 | 3 有一些 | 4 很多 | 5 全部都是 |
| 2. | 你屋企附近有1-3層的多個家庭住宅嗎（如村屋）？ | | | | |
|  | 1 沒有 | 2 很少 | 3 有一些 | 4 很多 | 5 全部都是 |
| 3. | 你屋企附近有4-6層的住宅嗎（如唐樓、私人樓宇等）？ | | | | |
|  | 1 沒有 | 2 很少 | 3 有一些 | 4 很多 | 5 全部都是 |
| 4. | 你屋企附近有7-12層的住宅嗎（如私人樓宇等）？ | | | | |
|  | 1 沒有 | 2 很少 | 3 有一些 | 4 很多 | 5 全部都是 |
| 5. | 你屋企附近有13-20層的住宅嗎（如公共屋邨、私人樓宇等）？ | | | | |
|  | 1 沒有 | 2 很少 | 3 有一些 | 4 很多 | 5 全部都是 |
| 6. | 你屋企附近有20層以上的住宅嗎（如公共屋邨、私人樓宇等）？ | | | | |
|  | 1 沒有 | 2 很少 | 3 有一些 | 4 很多 | 5 全部都是 |

**D．你屋企附近的環境**

請圈出最適合你的居住環境的答案。

| 1. | 我屋企附近的街道旁沿路有樹木。 | | | |
| --- | --- | --- | --- | --- |
|  | 1 非常不同意 | 2 有些不同意 | 3 有些同意 | 4 非常同意 |
| 2. | 我屋企附近沿途有很多有趣的事物可以看。 | | | |
|  | 1 非常不同意 | 2 有些不同意 | 3 有些同意 | 4 非常同意 |
| 3. | 我屋企附近有很多漂亮的自然景色（如花園、風景）可以看。 | | | |
|  | 1 非常不同意 | 2 有些不同意 | 3 有些同意 | 4 非常同意 |
| 4. | 我屋企附近有很多樓宇很漂亮。 | | | |
|  | 1 非常不同意 | 2 有些不同意 | 3 有些同意 | 4 非常同意 |
| 5. | 我屋企樓宇的平臺或天臺上有花園。 | | | |
|  | 1 非常不同意 | 2 有些不同意 | 3 有些同意 | 4 非常同意 |

**E．到達服務設施的便利程度**

當地和步行距離內均指從你屋企出發步行10-15分鐘可以到達的範圍。

| 1. | 從我屋企步行去商店很容易。 | | | |
| --- | --- | --- | --- | --- |
|  | 1 非常不同意 | 2 有些不同意 | 3 有些同意 | 4 非常同意 |
| 2. | 從我屋企出發，到許多地方都很容易（獨自或同他人）。 | | | |
|  | 1 非常不同意 | 2 有些不同意 | 3 有些同意 | 4 非常同意 |

**F．你屋企附近的街道**

| 1. | 我屋企附近的街道**沒有**很多掘頭路。 | | | |
| --- | --- | --- | --- | --- |
|  | 1 非常不同意 | 2 有些不同意 | 3 有些同意 | 4 非常同意 |
| 2. | 在我的屋企附近從一個地方去另一個地方有許多不同的路線，我不必每次都走同一條路。 | | | |
|  | 1 非常不同意 | 2 有些不同意 | 3 有些同意 | 4 非常同意 |

**G．行人設施**

請圈出最適合你的居住環境的答案。

| 1. | 我屋企附近的街道多數都有行人路。 | | | |
| --- | --- | --- | --- | --- |
|  | 1 非常不同意 | 2 有些不同意 | 3 有些同意 | 4 非常同意 |
| 2. | 我屋企附近的行人路與車路間有停泊的汽車分隔開。 | | | |
|  | 1 非常不同意 | 2 有些不同意 | 3 有些同意 | 4 非常同意 |
| 3. | 我屋企附近的行人路與車路之間有欄杆或花草。 | | | |
|  | 1 非常不同意 | 2 有些不同意 | 3 有些同意 | 4 非常同意 |
| 4. | 我屋企附近的行人路多數都有遮蓋。 | | | |
|  | 1 非常不同意 | 2 有些不同意 | 3 有些同意 | 4 非常同意 |

**H．你屋企附近的治安狀況**

請圈出最適合你的居住環境的答案。

| 1. | 我屋企附近的罪案率令**夜晚**外出散步（獨自或與他人）不安全。 | | | |
| --- | --- | --- | --- | --- |
|  | 1 非常不同意 | 2 有些不同意 | 3 有些同意 | 4 非常同意 |
| 2. | 我害怕獨自在屋企樓下逗留（如車道、住宅公共地方），因為我擔心被陌生人帶走或傷害。 | | | |
|  | 1 非常不同意 | 2 有些不同意 | 3 有些同意 | 4 非常同意 |
| 3. | 我害怕與朋友在屋企樓下逗留，因為我擔心被陌生人帶走或傷害。 | | | |
|  | 1 非常不同意 | 2 有些不同意 | 3 有些同意 | 4 非常同意 |
| 4. | 我害怕獨自或與朋友在屋企附近及周邊街道逗留或行走，因為我擔心被陌生人帶走或傷害。 | | | |
|  | 1 非常不同意 | 2 有些不同意 | 3 有些同意 | 4 非常同意 |
| 5. | 我害怕獨自或與朋友在附近公園逗留，因為我擔心被陌生人帶走或傷害。 | | | |
|  | 1 非常不同意 | 2 有些不同意 | 3 有些同意 | 4 非常同意 |
| 6. | 我屋企附近有令人覺得不安全的人出沒，如吸毒者、醉酒者、露宿者、或令人害怕的陌生人。 | | | |
|  | 1 非常不同意 | 2 有些不同意 | 3 有些同意 | 4 非常同意 |

**I．你屋企附近的交通安全**

請圈出最適合你的居住環境的答案。

| 1. | 附近街道夜晚的照明很好。 | | | |
| --- | --- | --- | --- | --- |
|  | 1 非常不同意 | 2 有些不同意 | 3 有些同意 | 4 非常同意 |
| 2. | 人們從家中很容易見到附近街道上的行人及踩單車者。 | | | |
|  | 1 非常不同意 | 2 有些不同意 | 3 有些同意 | 4 非常同意 |
| 3. | 屋企附近的街道上有斑馬線和紅綠燈幫助行人橫穿繁忙的道路。 | | | |
|  | 1 非常不同意 | 2 有些不同意 | 3 有些同意 | 4 非常同意 |
| 4. | 屋企附近的街道上有幫助行人過馬路的天橋或隧道 | | | |
|  | 1 非常不同意 | 2 有些不同意 | 3 有些同意 | 4 非常同意 |

**J．你屋企附近的空氣和噪音**

請圈出最適合你的居住環境的答案。

| 1. | 當在屋企附近行走時，有很多廢氣。 | | | |
| --- | --- | --- | --- | --- |
|  | 1 非常不同意 | 2 有些不同意 | 3 有些同意 | 4 非常同意 |
| 2. | 我屋企附近的街道上或商場外有很多人吸煙。 | | | |
|  | 1 非常不同意 | 2 有些不同意 | 3 有些同意 | 4 非常同意 |
| 3. | 我屋企附近的餐廳排出很多油煙。 | | | |
|  | 1 非常不同意 | 2 有些不同意 | 3 有些同意 | 4 非常同意 |
| 4. | 我屋企附近的地盤或工程發出很多噪音。 | | | |
|  | 1 非常不同意 | 2 有些不同意 | 3 有些同意 | 4 非常同意 |
