# Supplementary material for: Adaptation and evaluation of the neighborhood environment walkability scale for youth for Chinese children (NEWS-CC)
Source: BMC Public Health. 2021 Mar 11;21:480. doi: 10.1186/s12889-021-10530-4 (PMC7949240; doi:10.1186/s12889-021-10530-4)
Supplement: Supplementary file 2 — Additional file 2. [file 12889_2021_10530_MOESM2_ESM.docx]

**Neighborhood Environment Walkability Scale for Chinese Children (NEWS-CC)**

**A. Shops, restaurants and other public places in your neighborhood**

How long would it take you to walk from your house to the nearest shops or other places? Please circle one of the options, even if you do not usually go to the places.

| **1** | Convenience store (e.g.7-11) | 1-5 min | 6–10 min | 11-20 min | 21-30 min | 31+ min | don’t know |
| --- | --- | --- | --- | --- | --- | --- | --- |
| **2** | Supermarket | 1-5 min | 6–10 min | 11-20 min | 21-30 min | 31+ min | don’t know |
| **3** | Hardware store | 1-5 min | 6–10 min | 11-20 min | 21-30 min | 31+ min | don’t know |
| **4** | Public market | 1-5 min | 6–10 min | 11-20 min | 21-30 min | 31+ min | don’t know |
| **5** | Laundry/dry cleaners | 1-5 min | 6–10 min | 11-20 min | 21-30 min | 31+ min | don’t know |
| **6** | Clothing stores | 1-5 min | 6–10 min | 11-20 min | 21-30 min | 31+ min | don’t know |
| **7** | Post office | 1-5 min | 6–10 min | 11-20 min | 21-30 min | 31+ min | don’t know |
| **8** | Library | 1-5 min | 6–10 min | 11-20 min | 21-30 min | 31+ min | don’t know |
| **9** | Primary school | 1-5 min | 6–10 min | 11-20 min | 21-30 min | 31+ min | don’t know |
| **10** | Secondary school | 1-5 min | 6–10 min | 11-20 min | 21-30 min | 31+ min | don’t know |
| **11** | Bookstore | 1-5 min | 6–10 min | 11-20 min | 21-30 min | 31+ min | don’t know |
| **12** | Fast food restaurant (e.g. McDonald, Cafe de coral) | 1-5 min | 6–10 min | 11-20 min | 21-30 min | 31+ min | don’t know |
| **13** | Coffee shop (e.g. Starbucks) | 1-5 min | 6–10 min | 11-20 min | 21-30 min | 31+ min | don’t know |
| **14** | Bank/Finance center | 1-5 min | 6–10 min | 11-20 min | 21-30 min | 31+ min | don’t know |
| **15** | Non-fast food restaurant (Chinese restaurant/Tea restaurant) | 1-5 min | 6–10 min | 11-20 min | 21-30 min | 31+ min | don’t know |
| **16** | Video store | 1-5 min | 6–10 min | 11-20 min | 21-30 min | 31+ min | don’t know |
| **17** | Pharmacy | 1-5 min | 6–10 min | 11-20 min | 21-30 min | 31+ min | don’t know |
| **18** | Hair salon | 1-5 min | 6–10 min | 11-20 min | 21-30 min | 31+ min | don’t know |
| **19** | Any offices/ construction sites | 1-5 min | 6–10 min | 11-20 min | 21-30 min | 31+ min | don’t know |
| **20** | Mini bus stop/bus stop/MTR | 1-5 min | 6–10 min | 11-20 min | 21-30 min | 31+ min | don’t know |

**B. Leisure facilities and places in your neighborhood**

How long would it take you to walk from your house to the nearest places in the following list? Please circle one of the options, even if you do not usually go to the places.

| **1** | Indoor leisure or sport facilities (public or private) | 1-5 min | 6–10 min | 11-20 min | 21-30 min | 31+ min | don’t know |
| --- | --- | --- | --- | --- | --- | --- | --- |
| **2** | Beach, lake, river or creek | 1-5 min | 6–10 min | 11-20 min | 21-30 min | 31+ min | don’t know |
| **3** | Cycling/hiking/walking trail | 1-5 min | 6–10 min | 11-20 min | 21-30 min | 31+ min | don’t know |
| **4** | Basketball court | 1-5 min | 6–10 min | 11-20 min | 21-30 min | 31+ min | don’t know |
| **5** | Other playground/ sports field (e.g. soccer, rugby, baseball, tennis, skating) | 1-5 min | 6–10 min | 11-20 min | 21-30 min | 31+ min | don’t know |
| **6** | YMCA | 1-5 min | 6–10 min | 11-20 min | 21-30 min | 31+ min | don’t know |
| **7** | Youth Societies, e.g. The Boys’ & Girls Clubs Association of HK | 1-5 min | 6–10 min | 11-20 min | 21-30 min | 31+ min | don’t know |
| **8** | Swimming pool | 1-5 min | 6–10 min | 11-20 min | 21-30 min | 31+ min | don’t know |
| **9** | Jogging trail | 1-5 min | 6–10 min | 11-20 min | 21-30 min | 31+ min | don’t know |
| **10** | Schools with available sports fields open to the general public | 1-5 min | 6–10 min | 11-20 min | 21-30 min | 31+ min | don’t know |
| **11** | Sitting-out area | 1-5 min | 6–10 min | 11-20 min | 21-30 min | 31+ min | don’t know |
| **12** | Parks | 1-5 min | 6–10 min | 11-20 min | 21-30 min | 31+ min | don’t know |
| **13** | Children’s playground | 1-5 min | 6–10 min | 11-20 min | 21-30 min | 31+ min | don’t know |
| **14** | Open places (grass, sands, clay) | 1-5 min | 6–10 min | 11-20 min | 21-30 min | 31+ min | don’t know |

**C. Types of residential homes in your neighborhood**

“In your neighborhood” refers to the area within a 10-15 min walking distance from your own house

| **1.** | Are there any one-family residential homes (e.g. detached houses) in your neighborhood? | | | | |
| --- | --- | --- | --- | --- | --- |
|  | 1 None | 2 A few | 3 Some | 4 A lot | 5 All |
| **2.** | Are there any 1-3 storeys’ residential homes (e.g. villa, village houses) in your neighborhood? | | | | |
|  | 1 None | 2 A few | 3 Some | 4 A lot | 5 All |
| **3.** | Are there any 4-6 storeys’ residential homes (e.g. tenement house, private estates) in your neighborhood? | | | | |
|  | 1 None | 2 A few | 3 Some | 4 A lot | 5 All |
| **4.** | Are there any 7-12 storeys’ residential homes (e.g. private estates) in your neighborhood? | | | | |
|  | 1 None | 2 A few | 3 Some | 4 A lot | 5 All |
| **5.** | Are there any 13-20 storeys’ residential homes (e.g. public renting housing, private estates) in your neighborhood? | | | | |
|  | 1 None | 2 A few | 3 Some | 4 A lot | 5 All |
| **6.** | Are there any residential homes with more than 20 storeys (e.g. public renting housing, private estates) in your neighborhood? | | | | |
|  | 1 None | 2 A few | 3 Some | 4 A lot | 5 All |

**D. Neighborhood surroundings**

Please circle the answer that is most applied to your neighborhood.

| **1.** | There are trees alongside the streets in my neighborhood | | | |
| --- | --- | --- | --- | --- |
|  | 1 Totally disagree | 2 Somewhat disagree | 3 Somewhat agree | 4 Totally agree |
| **2.** | There are many interesting things to look at during walking in my neighborhood | | | |
|  | 1 Totally disagree | 2 Somewhat disagree | 3 Somewhat agree | 4 Totally agree |
| **3.** | There are many beautiful natural sceneries, e.g. garden and views, to look at in my neighborhood | | | |
|  | 1 Totally disagree | 2 Somewhat disagree | 3 Somewhat agree | 4 Totally agree |
| **4.** | Many buildings are beautiful in my neighborhood | | | |
|  | 1 Totally disagree | 2 Somewhat disagree | 3 Somewhat agree | 4 Totally agree |
| **5.** | There is a garden on the roof of the building I live | | | |
|  | 1 Totally disagree | 2 Somewhat disagree | 3 Somewhat agree | 4 Totally agree |

**E. Convenience of the accessibility to service facilities**

“within walking distance” refers to the area that you can reach by walk in 10-15 minutes

| **1.** | It is easy to walk from my house to the shops. | | | |
| --- | --- | --- | --- | --- |
|  | 1 Totally disagree | 2 Somewhat disagree | 3 Somewhat agree | 4 Totally agree |
| **2.** | It is easy to go to various places from my house in my neighborhood (alone or with accompany). | | | |
|  | 1 Totally disagree | 2 Somewhat disagree | 3 Somewhat agree | 4 Totally agree |

**F. Streets in your neighborhood**

Please circle the answer that is most applied to your neighborhood.

| **1.** | There are **NOT** many cul-de-sacs in my neighborhood. | | | |
| --- | --- | --- | --- | --- |
|  | 1 Totally disagree | 2 Somewhat disagree | 3 Somewhat agree | 4 Totally agree |
| **2.** | There are many different routes from one place to another in my neighborhood so that I don’t have to walk the same route every time. | | | |
|  | 1 Totally disagree | 2 Somewhat disagree | 3 Somewhat agree | 4 Totally agree |

**G. Sidewalks**

Please circle the answer that is most applied to your neighborhood.

| **1.** | Sidewalks are available on most of the nearby streets in my neighborhood. | | | |
| --- | --- | --- | --- | --- |
|  | 1 Totally disagree | 2 Somewhat disagree | 3 Somewhat agree | 4 Totally agree |
| **2.** | Vehicles parked on the nearby streets separate the sidewalks from traffic lanes in my neighborhood. | | | |
|  | 1 Totally disagree | 2 Somewhat disagree | 3 Somewhat agree | 4 Totally agree |
| **3.** | Barriers or grass separate the sidewalks from traffic lanes in my neighborhood. | | | |
|  | 1 Totally disagree | 2 Somewhat disagree | 3 Somewhat agree | 4 Totally agree |
| **4.** | Most nearby sidewalks have covers. | | | |
|  | 1 Totally disagree | 2 Somewhat disagree | 3 Somewhat agree | 4 Totally agree |

**H. Crime issues in your neighborhood**

Please circle the answer that is most applied to your neighborhood.

| **1.** | Crime rate in my neighborhood make it unsafe to walk outdoors (alone or with accompany) in the evenings. | | | |
| --- | --- | --- | --- | --- |
|  | 1 Totally disagree | 2 Somewhat disagree | 3 Somewhat agree | 4 Totally agree |
| **2.** | I am afraid of being outside alone near my house in my neighborhood, e.g. streets, public places, because I am worried about being taken or hurt by a stranger. | | | |
|  | 1 Totally disagree | 2 Somewhat disagree | 3 Somewhat agree | 4 Totally agree |
| **3.** | I am afraid of being outside with my friends near my house in my neighborhood, e.g. streets, public places, because I am worried about being taken or hurt by a stranger. | | | |
|  | 1 Totally disagree | 2 Somewhat disagree | 3 Somewhat agree | 4 Totally agree |
| **4.** | I am afraid of being outside or walking near my house or on the streets in my neighborhood (alone or with accompany), e.g. streets, public places, because I am worried about being taken or hurt by a stranger. | | | |
|  | 1 Totally disagree | 2 Somewhat disagree | 3 Somewhat agree | 4 Totally agree |
| **5.** | I am afraid of being outside alone or with my friends in the nearly parks in my neighborhood because I am worried about being taken or hurt by a stranger. | | | |
|  | 1 Totally disagree | 2 Somewhat disagree | 3 Somewhat agree | 4 Totally agree |
| **6.** | There are people who make me feel unsafe in my neighborhood, such as drug abuser, inebriates, homeless people, and dreadful strangers. | | | |
|  | 1 Totally disagree | 2 Somewhat disagree | 3 Somewhat agree | 4 Totally agree |

**I. Traffic safety**

Please circle the answer that is most applied to your neighborhood.

| **1.** | The nearby streets are lit very well in the evenings. | | | |
| --- | --- | --- | --- | --- |
|  | 1 Totally disagree | 2 Somewhat disagree | 3 Somewhat agree | 4 Totally agree |
| **2.** | It is easy to see pedestrians and cyclists on the nearby streets from the houses in my neighborhood. | | | |
|  | 1 Totally disagree | 2 Somewhat disagree | 3 Somewhat agree | 4 Totally agree |
| **3.** | There are crosswalks and signals to help people cross the busy streets in my neighborhood. | | | |
|  | 1 Totally disagree | 2 Somewhat disagree | 3 Somewhat agree | 4 Totally agree |
| **4.** | There are bridges or tunnels to help people cross the busy streets in my neighborhood. | | | |
|  | 1 Totally disagree | 2 Somewhat disagree | 3 Somewhat agree | 4 Totally agree |

**J．Air and noise pollutions**

Please circle the answer that is most applied to your neighborhood.

| **1.** | There is much exhaust gas when I walk on the nearby streets in my neighborhood. | | | |
| --- | --- | --- | --- | --- |
|  | 1 Totally disagree | 2 Somewhat disagree | 3 Somewhat agree | 4 Totally agree |
| **2.** | There are many smokers in the streets or outside the shopping centers in my neighborhood. | | | |
|  | 1 Totally disagree | 2 Somewhat disagree | 3 Somewhat agree | 4 Totally agree |
| **3.** | There is much fume from nearby restaurants. | | | |
|  | 1 Totally disagree | 2 Somewhat disagree | 3 Somewhat agree | 4 Totally agree |
| **4.** | There is much noise from nearby worksites. | | | |
|  | 1 Totally disagree | 2 Somewhat disagree | 3 Somewhat agree | 4 Totally agree |
